# The Natural Defense

**Published:** 1918-09

**Authors:** 


### THE NATURAL DEFENSE

The mere fact that man is surrounded, covered, and penetrated by an infinite number of bacteria and lives, is a proof that the human body has within itself adequate defense against bacteria. This defense we may presume was attained through struggle and survival — that is, through biologic adaptation, — and is the bulwark of the treatment of infection.

It is an interesting fact that the various parts of the body are endowed with differing degrees of power of defense. In general, the parts most exposed to infection by reason of injury, such as the external soft parts; or by reason of being in an infecting neighborhood, such as the intestine, the parts of the body exposed to injury and those exposed to infected areas are endowed with a mechanism for an efficient defense.

On the other hand, the inner protected parts — such as bone, especially bone deeply placed; and deep muscles and protected organs and tissues, such as the heart, the brain, the spinal cord, the retroperitoneal tissue and fat — have had less opportunity to make a selective struggle, hence have weaker defenses. What is possessed by the tissue of the face that is not possessed by the mediastinum, or the femur? What is possessed by the peritoneum that is not possessed by the dura that endows them with a better defense? The part with weakest defense usually shows by comparison a limited blood supply, and when infected the reaction is mild, and is lacking in both the appearance and the fact of good defense. There is apparently no other difference as striking nor as marked as the difference in blood supply, either normally or in reserve, which may be brought out by infection. We may then infer that the means of defense is mainly in the blood.

A rich blood supply is then the key to the defense. The face and scalp, the external parts, and the abdomen, have a rich supply as compared with the deep lumbar muscles, the bone, the spinal cord, the retroperitoneal tissue. Not only is the normal blood supply less in these poorly defended regions, but the local vaso-motor mechanism is less developed, hence there is less reaction. For the highest defense there must be not only abundant blood, but normal blood. The bones, the deep tissue planes, the mediastinum, the spinal cord, the brain, the retroperitoneal space are all hazardous regions for infection. The surgeon should have in his mind the body charted like the sea, and should sail according to his chart. One region requires this conception, that plan; another, such as the scalp and face, no special plan. Face wounds heal well with good surgery — even with no surgery. The infected mediastinum, with no surgery, with poor surgery, with good surgery, does almost equally badly. A pulseless patient becomes a universal mediastinum. A limb anemic — as from a neglected tourniquet — from severing of arterial supply, becomes as helpless as the meninges; the patient in exhausion from cold and wet and exposure, and loss of sleep and fatigue from fighting, has a universally weak defense. Patients prostrated by shock or by hemorrhage have low resistance. As a rule, defeated, dejected troops have less resistance than victorious troops. The defense, then, in the normal soldier varies with the several parts of the body, the frontiers of the organism being best defended. The defense, even at the frontiers of the organism, is lowered by interference with the local blood supply, whether of an entire limb, as strated, or of the devitalized bloodless tissue along the injured track of the missile; or of low blood-pressure from shock, with its secondary acidosis; or from hemorrhage; or when the entire individual is in exhaustion. The defense in turn is augmented by rest, sleep, fluids and revision of wounds, and by restoration of the local and the general blood supply. Excision of devitalized tissue, rest, a night's sleep, a transfusion of blood become valuable "antiseptics" and tend to restore and preserve the natural defense.

